# Flexible learner or imposter? Learning A Level mathematics in England through the COVID-19 pandemic

**DOI:** 10.1093/teamat/hrab025

**Published:** 2021-10-26

**Authors:** Jennie Golding

**Affiliations:** University College London Institute of Education, London WC1H0AL, England

## Abstract

We evidence English teacher and student perspectives on the learning of pre-university mathematics ‘A Level’ courses through the pandemic period to July 2021. Data are drawn from a 2017–21 classroom-close study of enactment of such courses in 13 fairly representative centres, using an institutional ethnographic approach. The pandemic picture was generally one of the significant and sustained negative impacts, though over the course of the study, respondents reported progress in addressing early limitations in the harnessing of digital platforms for learning. A small number of participating students reported home-based study beneficial for their mathematics learning, and a bigger group identified some wider benefits that partly offset the challenges. Most participating 16–18-year-old students, though, reported finding remote learning of mathematics both demanding and limiting. Pandemic constraints impacted most strongly on opportunities to engage with newer emphases within A Level courses: problem solving, reasoning, modelling, statistics and mechanics. Receiving academics reported that mathematical preparedness, and confidence, for mathematics-intense university courses has also been widely affected, with a bigger range of preparedness and confidence than usual. The study draws attention to the importance of studying subject-specific impact and drawing on student as well as teacher perceptions. It exposes a range of consequences of the cancellation of examinations and a need to develop and share effective pedagogies for working remotely with pre-university students.

## 1 Background

### 1.1. The pandemic and mathematics learning

In England and more widely since early 2020, there have been considerable efforts to understand the impact of the coronavirus pandemic on learners, though largely in generic terms (e.g. Eivers *et al.*, 2020; Institute for Fiscal Studies, 2020). As a key subject, progression in mathematics-specific attainment, even if somewhat coarsely assessed, has received some attention. In England, generic studies have found that teachers were often professionally ill-equipped to pivot to remote teaching. Individual schools’ responses have diverged, and students’ engagement with learning opportunities offered has also varied considerably, often exacerbating existing underprivilege. There has also been some small-scale mathematics-focused work (e.g. Hodgen *et al.*, 2020; Royal Society and Joint Mathematical Council, 2021; Drijvers *et al.*, 2020). Such studies complement pre-existing evidence on school closure periods and on fulltime remote learning (e.g. Gill, 2015). Earlier evidence pre-dates recent progress in access to digital technologies for learning. The reported study is therefore distinctive in offering mathematics-specific, digitally imbued, ‘stories’ of pandemic impacts on students approaching transition to university.

One aspect of a sudden move to remote teaching and learning is that it makes considerable demands on teachers: digital approaches to teaching and learning require both a facility with technology and a capacity to support students with that (Mishra & Koehler, 2006). They also need a capacity to transfer and adapt face-to-face mathematics teaching capacity to the new medium, redesigning familiar teaching approaches for remote (synchronous or asynchronous) use (Smith & Bretscher, 2018; Ruthven, 2009). However, at a pre-university level in England, sustained and systemic use of remote digital teaching and learning had not in March 2020 been well developed. Many teachers were not well equipped or pedagogically or technologically knowledgeable about moving their teaching online (Ofsted, 2021). It might be reasonable to assume that most 16–18 year olds in an affluent country, the subjects of this study, would be ‘digital natives’, as well equipped and mature as any school-age students to engage with, and possibly benefit from, the affordances of those technologies. However, students, also, need not only access to and fluency with the technology used: even more than in face-to-face learning, they depend on intrinsic motivation and self-regulation for learning (Education Endowment Fund, 2020).

### 1.2. The policy background

In England, the most common pre-university qualifications for years 12 and 13 are ‘A Levels’, with Mathematics A Level the core calculus-focused course for students wanting to use mathematics at university. That is supplemented by ‘Further Mathematics’ A Level for those with more focused mathematical interests or aspirations. A Level teaching is largely based in 11–18 schools or post-16 colleges, referred to as ‘centres’. Mathematics A Level remains the single most popular A Level in England, attracting students with a relatively high average prior attainment (Joint Council for Qualifications, 2020).

A Level qualifications are high stakes for students. Related assessment materials and curriculum materials operate in a marketplace, and for mathematics A Level qualifications and materials the market leader is Pearson. It is therefore important for Pearson to evaluate the efficacy of their products, and this paper reports part of a Pearson-funded 2017–21 study focused on the most recent A Level specifications. These were first taught from September 2017 and feature a renewed focus on mathematical problem solving and proof. They require all students to study mechanics and statistics, including engagement with a large dataset using technology. The reported study asked

- *How are Pearson mathematics A Levels resources and assessments used with and by students and teachers? What is the impact of that on classroom experiences and the range of valued student outcomes, including appropriate progression?*

Study data from centres using Pearson resources up to March 2020 suggested that the new requirements have been challenging for both students and teachers (Mason *et al.*, 2021). Over half of the study teachers said that they felt poorly prepared to support learning of those by the range of A Level students and ill-equipped to teach the use of technology required for the large data set work. Overall, at least 60% of years 12 and 13 students and their teachers reported significant pressure on time and performance in studying for the new mathematics A Levels. This in some cases caused loss of student confidence and respondents reported these pressures have the potential to lower future participation rates.

In March 2020, data collection was interrupted by the onset of the coronavirus pandemic. All centres were closed to most students from mid-March to July 2020, and again January-early March 2021. All students could return to face-to-face (but pandemic-constrained) teaching for September–December 2020 and again from March 2021, except for virus-related periods of teacher or student isolation. The focus study was adapted to answer

- *How have practice in, learning of, and outcomes from, mathematics A Levels developed since the onset of the pandemic in 2020?*

This paper focuses on the answers to that research question from March 2020 to July 2021. Enquiry included consideration of the impacts of the ‘centre-assessed grades’ used in Summer 2020 and the ‘teacher-assessed grades’ used in Summer 2021, as substitutes for the usual terminal examinations. The paper contributes mathematics-specific stories of pandemic impacts on three successive cohorts of students approaching transition to university.

### 1.3. The study

The wider study uses an institutional ethnographic approach (Smith, 2005) to solicit data from 13 centres reasonably representative in terms of A Level mathematics outcomes, student prior attainment, governance and size and external inspection grade, though there is little claim to easy generalizability. Because of Pearson’s market leadership, the findings are likely to extend widely. Within each centre, we accessed Head of Mathematics’, mathematics A Level teachers’, and years 12 and 13 mathematics A Level students’ perspectives, focusing usually on one year 12 and one year 13 mathematics A Level(s) class that we followed through their A Level course(s).

**Figure 1:**
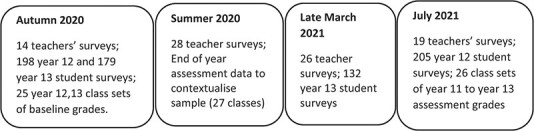
In-pandemic mathematics A Level study data collection events.

Our approach has resulted in large-scale data collection but importantly, sustained working relationships with teachers and students in the sample centres that laid foundations for continuing in-pandemic research. From late March 2020, all data collection had to move online, but building on such relationships, resulted in the data summarized in Fig. 1:

We also had access to 218 year 13 student surveys completed during March 2020 over the introduction of the first lockdown. All surveys were semi-structured, consistent with an ethnographic approach, and resulting in generally unstructured responses to each question. Many responses were both sustained and reflective, offering rich data, and we hypothesize the related participant generosity arose at least in part because of the working relationships already established. Online survey data, as with other survey data, are often superficial and brief. Our pandemic-related data are therefore unusual in their richness. Brief reports of some aspects of our findings are reported in Redmond *et al.* (2020, 2021), but here we focus on an overview of the mathematical ‘journeys’ of the A Level cohorts most immediately affected by the pandemic.

We draw also on a parallel element of the study (summarized in Mason *et al.*, 2021), which in Autumn 2020 explored student progression into mathematics-intensive university courses from mathematics A Levels by probing academics’ perceptions of the mathematical foundations brought to university by first-year students entering in each of Autumn 2019 and Autumn 2020. There was no direct student input into this element. Data comprised 44 responses, via interview or similar questionnaire, from a purposive sample of academics from a range of 34 different universities in England, 16 of them the most selective ‘Russell Group’ universities: 31 academics whose work is focused on mathematics, including first year undergraduate mathematics and 13 academics whose work includes first-year applications of mathematics (mathematical economics, physics, engineering and study support for the range of mathematics users). Selected findings from that work ‘complete’ the pandemic story in relation to students in year 13 when the pandemic took root.

Throughout all parts of our work, qualitative data were analysed first by research sub-question, then, using a grounded approach (Charmaz, 2006) through open, axial and thematic coding within each of those, by the UCL/Pearson research team. For example, one theme was ‘medium-term impacts of the pandemic’, with sub-themes ‘mathematics participation and progression’, ‘embedded mathematical skills, knowledge, processes’ and ‘student aspirations and attitudes’ and reporting featured copious related quotations to support a ‘thick description’ of student and teacher experiences. This paper focuses on findings that relate to impact of the pandemic, although it also situates those in wider findings. Ethical approval was from the author’s university, and the author’s institutional mathematics education research group validated the approaches to design, analysis and interpretation.

Steps taken to support research integrity, including in relation to conflict of interest with the funder, included the lead researcher (the UCL author) having access to all data no later than any Pearson employee, cross-researcher validation of at least 10% of coding, with all differences negotiated and recent validation achieving over 97% initial agreement, occasional participant validation of interpretation where this was unclear (though not with student participants since those were anonymous for ethical reasons) and cross-researcher triangulation of grounded emergent themes and of writing. Use made of the research by Pearson (e.g. Mason *et al.*, 2021) was also validated, and sometimes challenged, by the lead researcher. Throughout detailed internal technical reports, typicality/extent of reported responses and impacts was quantified and selection, including of quotations, made reflexively and with co-researcher validation; all other papers and communications are derived from those internal reports, with checking of original data at key points. Teacher and academic participants have had the opportunity to validate emergent accounts.

So what changes did the pandemic bring for mathematics A Level students? Drawing on the in-pandemic data collected to April 2021, we consider in turn the evidence for the ‘core’ (year 12 into 13) A Level cohort, for those transitioning into A Levels from year 11 during this period and for those transitioning from year 13 into mathematics-intensive university courses, since these three cohorts had distinctive sets of experiences. In what is normally a ‘high stakes’ examination system, we then analyse the data relating to examinations and to the ‘centre-assessed grades’ that replaced them in Summer 2020, as well as pre-publication responses to the ‘teacher-assessed grades’ used in Summer 2021.

## 2 Findings

### 2.1. Experiences of the core A Level cohort

A Levels are studied over years 12 and 13. The 2019–20 year 12 students we refer to here as the ‘core A Level cohort’ relative to the pandemic to date, as their A Level learning was impacted from March in year 12 through to the end of their course in June 2021. The preceding cohort usually transitioned into university in September 2020, and the succeeding cohort transitioned into A Levels at the same time. For the core cohort, most teachers were confident teaching and near-normal pace of expected coverage had been maintained from March–July 2020, though synchronous provision had been unusual. Very often year 12 students in the core cohort had experienced between an hour and 2 weeks back in centre prior to the summer holidays, though at least 70% of responding teachers reported they had only poor evidence of the March–July target student learning. There was a related cost:

*Most students found it very hard to work independently and we couldn’t monitor what they were doing or how. Consequently their mathematical knowledge and skills have declined. (Teacher 1, Centre 6).*

By late Autumn 2020, the then-year 13 responding core cohort students (*n = 179*) reported having experienced a variety of (sometimes significant) and continuing pandemic-related challenges to their learning and to their personal lives, although occasionally also, some benefits. A large majority (over 90%) had found it very difficult to learn effectively remotely, with concentration, feelings of isolation and understanding reported to be key issues: ‘*A good cry every now and again and watching youtube videos on topics I struggle with’ (y13 Student 109);* ‘*I feel that firstly I hadn’t understood most of the lockdown work and secondly I had forgotten most of the previous work’ (y13 Student 30); ‘Stopped my learning, nothing got done in terms of school work at home, I couldn’t concentrate’(y13 S10); ‘It was easy to feel isolated in lockdown, so talking to peers became difficult’ (y13 Student 35).*

Students often (at least 42%) thought teachers had targeted more routine and superficial learning during the pandemic and particularly when working remotely: ‘*When online there is only ever surface level coverage, we don’t do hard bits like proof parts of a question’ (y13 Student 9)*—but had still struggled with that. Many students (at least 58%) had significant concerns about an impoverished mathematical diet for their progression, though the difference from a ‘normal’ cohort, at this time of year, is unclear. A small minority (5%) felt the home learning period had suited their preferred approaches to learning, personality or body clock, and that they had gained from the flexibility available, and the opportunities to revisit or pause recorded resources. Some (4%) also analysed that the digital and independent learning skills developed would be well used in independent work at university and beyond: ‘*I have developed an aptitude for self learning and discipline, that will stand me in good stead next year’ (y13 Student 34);* ‘*Ability to focus on my weakness and tailor my learning to overcome these: good prep for uni’ (y13 S 94);* ‘*I can rewind videos teachers record and learn at my own pace’ (y12 S20).*

‘Remote’ learning does not equate to digital learning, and indeed, the majority of students (69%) chose to work from printed textbooks rather than the digital versions usually also available to them. However, 10% reported inadequate access to internet or digital device for their learning needs: ‘*I share computers with my siblings who are also in remote learning. When I find an alternative (device) my mic/camera are unable to work although I can still type and listen’ (y13 Student 157).*

By March 2021, most students (62%) in this cohort thought they were gaining in confidence to work remotely, but over half reported the structure of the work required often had little variety, and/or only slowly developing teacher use of the affordances of digital platforms for groupwork, display, formative and summative assessment, etc. For teachers thrown suddenly into remote learning provision, such conservatism is unsurprising. Six students of 179 said they thought their mathematical confidence had been further dented by the lack of Summer 2020 internal examinations, which would have catalysed a synthesizing of learning and given an objective measure of progress: some students (at least half) in those three centres which had managed to run internal assessments in some way appreciated that opportunity.

In late Autumn 2020, almost all responding core cohort students, now in Year 13, said they felt in-centre learning was significantly easier than learning remotely, though most (over 70%) felt they were still under-performing compared with potential. In-centre pace and depth were typically (in 64% of responses) reported much-reduced from ‘normal’, except in the most academically selective centres—and less ‘hands-on’, as teachers tried to ensure foundational knowledge but also teach for timely ‘coverage’, while simultaneously physically constrained in the approaches they could use. In centre, students were typically (at least 58%) further constrained by reduced access to teachers, to peer group working and to independent study space when in centre, as well as by additional periods of student or teacher self-isolation: ‘*It is difficult, no group work to study in, no access to library’ (y13 Student 15).* Accumulated learning gaps from the home learning period in year 12 were a significant issue for these year 13 students, many of whom (at least 32 of 179) reported having been able to learn only superficially: ‘*We have only a base level understanding of the content rather than a full in depth understanding’ (y13 Student 105).* Additional structures such as submitted homeworks, well-structured, challenging lessons and in-support opportunities back in centre and end of year internal examinations were reported helpful to restoring mathematical confidence.

In terms of overall approach to the A Level, students reported little use of mathematics-specific software for learning, either remotely or on return to centre. Within mathematics, problem solving, proof and applied areas were thought to be most challenging to develop remotely (nearly 70% of students mentioned at least one of these), but those areas were also being marginalized in face-to-face teaching, partly in an effort to ensure ‘coverage’: ‘*Haven’t got enough depth of knowledge to tackle problem solving questions: we’re focusing on covering the syllabus’ (y13 Student 80)*;

*Mechanics (is hard) as there was no actual lessons for me to see and understand the modelling, it was always from a book. Mechanics is essentially practical, and there’s a particular way of thinking about it that it’s hard to “catch” from a book (y13 Student 123).*

23% of participating students, though, reported they had found remote means of working constructively with peers: *I created a study group where we would all join a Teams meeting and do maths questions and topics…. (That) helped all of us in revising and maintaining productivity (y13, S107).*

At least 71% of teachers in late Autumn 2020 felt year 13 attainment had been negatively affected, though not at that point irretrievably so; some (36%) were still challenged to assess the degree of learning loss. Students who had not engaged in depth in Summer 2020 were thought most likely to be vulnerable to progression difficulties. Such issues were less obvious in the two academically selective centres.

Years 12 and 13 students (the core cohort and their successors) identified reduced opportunities for practical work, enrichment, work experience and university visits as important impacts of the pandemic, and those were reported to threaten confidence to progress as planned: ‘*I want to go university, and study pharmacy. But I think the pandemic have impacted a lot because I can’t showcase my full potential through placements (y13 Student 67);* ‘*Due to the pandemic it has been extremely difficult to find work experience, which is crucial when applying for medicine, so I’m not confident about offers’ (y12 S3).* However, while reduced attainment seemed a real threat to year 13 students, the ~third of students who reported changes to planned routes typically said challenges to attainment resulted in them applying for less ambitious pathways in the same field (e.g. a less highly rated university), with only one student reporting a change to their intended area of further work or study. It is not clear how this compares with year 13 student behaviour in more ‘normal’ circumstances.

Importantly, many students (over 26%) reported digital marginalization of some sort, and this was somewhat correlated with the socio-economic status of the centre catchment area: ‘*As I did not have internet, peer support and socialising was difficult and added to stress and worry’ (y13 Student 150); ‘Wifi was an issue had to use my own data. My laptop is old so it was very slow I had to keep going over the lessons and recording’ (y13 Student 81).* Others, across centres (at least 5%), said they have suffered, and often continued to suffer, significant mental health, emotional and security issues associated with the pandemic: ‘*Not much progress, mental stability isn’t well’ (y13 Student 67);*

*I think so many students, including me suffered extreme loss over this period, not only grieving people but grieving a life we feel like we’ve lost, friends we used to see every day we just can’t anymore and it’s so, so hard… I don’t think exam boards really are taking into account the actual mental stress of this year and have expected us to not only teach ourselves content but also cope like normal under these conditions (y13 S105);*

Following Autumn 2020 data collection, A Level students then experienced a further 2-month period of home learning, before returning to in-centre learning in early March 2021. Both students and teachers later reported significantly enhanced variety, structure and demand of remote learning over the renewed lockdown. At least 88% of responding teachers claiming significantly enhanced confidence to select and structure remote teaching, to teach ‘live’ lessons (although those also brought challenges of bandwidth in many homes), and to harness a wider range of affordances of digital platforms, compared with the first such period. Only 3% of responding students reported difficulties in accessing appropriate hardware in early 2021, although 17% reported repeated challenges with internet access. However, at least 85% of teachers, and 88% of students, reported considerable, persisting, difficulties in teaching or learning particular aspects of the A Levels, including problem solving, proof, mechanics and some aspects of statistics; work with the large data set was reported further marginalized compared with pre-pandemic. Reduced access to teachers, and to peer groups, remained key concerns for a wide range of students, together with uncertainties about future pathways and preparation for those. Associated with this range of concerns were reports (from at least 23% of teachers and 37% of students) of student mental health issues attributable to, or exacerbated by, the pandemic. However, both teachers and students showed enhanced awareness of potential student gains from learning remotely—from reduced travelling time, to enhanced confidence and fluency in learning independently.

### 2.2. Transition from year 11 into A Levels during the pandemic

The second A Level cohort we consider is those students who were in March 2020 in year 11, expecting to take summative examinations in May/June 2020 prior to embarking on A Level courses. In March 2020, teachers suddenly had to provide a structure for distance learning. Our data suggest teachers in a majority of centres understandably prioritized year groups who were mid-course rather than those who had nearly completed their courses, particularly following announcement of the use of centre-assessed grades based on existing assessments. In common with other studies (e.g. Institute for Fiscal Studies, 2020), our data showed only a small proportion (less than 13% of our teacher respondents) expected post-closure work from all year 11 students. Others (41%) said they focused on those students planning on taking Mathematics A levels, while some (46%) expected no work from any year 11 s. However, the March–June period is typically a particularly constructive one of synthesis and consolidation of mathematics learning for year 11 students (Flintcroft *et al.*, 2017), so some of these approaches are likely to have resulted in considerable loss of the usual learning.

In December 2020, teachers did indeed report sizeable insecurities in the prior learning of year 12 students, widely attributed to limited synthesis and consolidation of earlier learning, along with, for many, a nearly six-month gap in academic work. In late 2020, teachers typically anticipated such gaps were likely to be recoverable over the 2-year course: ‘*I expect the yr12s to catch up over the rest of the year, as long as the students who need to catch up do not miss school for another prolonged period’ (Teacher 2, Centre 4)—*although of course the early 2021 lockdown followed.

Many students in this cohort (74% of responding Year 12 students in December 2020) reported very little achieved engagement with mathematics from March to September 2020. Although about half had been *offered* work from their school, over 70% of those reported that the uncertain and unsettling nature of the period had meant motivation had been poor, and even those who did engage often found it difficult to focus on deep learning. Without exception, students reported valuing in-centre attendance in Autumn 2020, though their learning continued affected by physical constraints, and teacher and student periods of isolation: ‘*It’s very hard to focus and not do something else, and it’s kind of one-D just using a laptop all the time, when everyone else is in college’ (y12 Student 24).*

As with the core cohort, 72% reported usually focusing on core knowledge and skills rather than more demanding processes such as problem solving and reasoning, and practical and groups work were severely constrained. A total of 63% expressed concerns about the robustness of their mathematical progression as a result, and some limitations to confidence because progression had not been based on examinations, while at least 31% of responding students were frustrated at limited pace while teachers ensured the robustness of mathematical building blocks. However, by December 2020 students typically appeared reasonably confident gaps could be addressed over time. In other respects, these year 12 students’ experience of transition into A Levels seemed no more problematic than in our Autumn 2017–19 data, perhaps because, as our Summer 2020 data show, teachers had anticipated this cohort needing enhanced support. As with the ‘core’ cohort discussed above, there was some evidence that pandemic constraints on learning were having less impact on some students with the highest prior attainment, or in historically high-attaining centres.

In July 2021, despite considerable persistent concerns evidenced by teachers, data from the 204 responding students in this cohort evidenced a remarkable resilience to the challenges to learning encountered, even when that had included further periods of student or teacher covid-related isolation and so remote learning. By July 2021, many year 12 students still had significant concerns about impacts on their learning, and on their future pathways, in line with previous data, but were generally more phlegmatic about that than the preceding cohort of students had been. They were also more active in identifying positive impacts on independent and remote learning and on their future trajectories—perhaps having seen the previous two year groups’ progress successfully beyond A Levels. However, technical marginalization of a minority of students remained a significant concern for both teachers and students.

### 2.3. Transition into university

The final cohorts we consider are those who in March 2020 were already in year 13. Our data suggest that once external examinations had been cancelled, little monitored work had been expected from year 13 in most (77% of) centres, especially after all topics had been encountered: teachers understandably prioritized other year groups. They sometimes (in at least 46% of centres) suggested how year 13 students might helpfully take their work forward but did not monitor response. Consequently, in July 2020 respondent teachers expected a significant impact on the cohort’s learning, likely to be felt especially if students were intending to use A Level mathematics skills and knowledge in their university course. However, by December 2020, these teachers had little information from former students.

The university-focused element of our study showed that receiving academics widely (at least 64% of respondents) considered the mathematical foundations of the 2020 entry cohort to be markedly weaker, on average, than their predecessor cohort’s, and with greater range of initial functioning and preparedness—across the categories of mathematical preparedness probed, and all levels of A Level grades awarded. Within students’ mathematical preparedness, academics expressed greatest confidence in students’ basic knowledge, and application in their study area of mathematical facts and of core standard processes, which were on average considered near-adequate. Data handling and mathematical problem solving and reasoning within their study area were typically considered ‘weak’ to ‘adequate’, while students’ ability to model mathematical situations, to engage with unfamiliar mathematical situations, to reason more abstractly and to communicate mathematically was an area for significant concern. In all respects, these findings compared unfavourably with those in relation to the 2019 entry.

Further, 2020 entry students were reported often ill-equipped to cope with pressures of university assessments, with less confidence, and greater incidence of unresolved ‘imposter syndrome’:

*Lack of confidence. In 2019 when students lacked confidence or had ‘imposter syndrome’ we could point to their exam results and say that they deserved to be here. This year, there is no such concrete reinforcement of their abilities (in their opinion) (Survey academic 23).*

Imposter syndrome is known to correlate with under-represented groups, including gender (Canning *et al.*, 2020), so this finding raises equity and inclusion issues. Students had reported that pre-university examinations were a good motivational tool for teaching and learning. Over half of participating academics pointed to examinations’ role in supporting focus on synthesis and contextualization of learning, which incoming students reported more key in mathematics than in other school subjects. As a consequence,

*I think they were a little more rusty on the recalling facts/ procedures, that traditionally most new students are good at – they didn’t have to synthesise their learning to the same extent in the summer (Survey academic 24).*

Students themselves also commonly reportedly identified that as a result of examination cancellation, they had a weaker repertoire of core knowledge and processes, as well as a more superficial synthesis of their school mathematics.

However, academics also reported some small signs that students might have benefited from online and then blended mode of experiences, perhaps in terms of independent approaches to learning of less structured course elements such as coursework. Students themselves had identified enhanced organization for learning as a benefit. While the data suggest that many universities could perhaps usefully revisit their approach to supporting transition, these responses from academics do very much point to what might be missed when the usual structures and roles of examinations are suddenly removed. Teachers and students in centres offered supplementary data relating to perceived impact on learning of the use of centre- or teacher-assessed grades in place of external examinations, and it is to those we now turn.

### 2.4. Impact of centre-assessed and teacher-assessed grades

2020–21 Year 12 students, the second cohort considered, had experienced use of centre-assessed grades (CAGs) in year 11. These were largely based on centres’ existing assessment data, moderated by a variety of teacher and managerial inputs that differed between centres. Some reports of perceived ‘unfair’ calculation of CAGs were strongly expressed, though only 2% of responding students thought their progression to year 12 had been directly impacted. At least an additional 4% thought the grades awarded might later have an adverse impact on their progression to university. Looking forward given their continuing-disturbed teaching in year 12, about a third of students thought standard A Level examinations were the ‘fairest’ method of assessment despite learning having been lost, with some concerned about loss of credibility from CAGs. Another third thought that in similar circumstances in the future, focused teacher-run assessments would be preferable and often argued that such an approach supported steady application through the course. The remainder had more mixed views, with some proposing innovative solutions that combined teacher assessment and examinations.

CAGs as originally proposed for June 2020 were generally thought by teachers to be the ‘least bad’ solution although some thought retaining at least A Level examinations would have been fairer; for CAGs there was concern about late-developing students and inter-centre comparability. The opportunity to take examinations in Autumn 2020 instead was valued. Teachers had mixed views about the reliability and validity of Summer 2020 CAGs:

*It is always going to be both honest and dishonest CAG’s. Some teachers are not very experienced either. I experienced it with other colleagues especially in GCSE predictions (Teacher 1, Centre 8).*

About a third of year 13 students thought CAGs of some sort would be the fairest approach for Summer 2021, and another third felt strongly that examinations were fairer and more credible. The remainder were more equivocal, recognizing CAGs would be unlikely to ‘work’ in the same way a second time and making a variety of suggestions, including modification of exam expectations or a combination of CAGs and examinations: ‘*Perhaps use a mix: exams are the only really fair way, but they could perhaps have a contribution from teachers’ (y12 Student 170);* ‘*The fairest would be to do the exams but with a more generous grading system so that we are not outcompeted by last year’s cohort’ (y13 Student 128);* ‘*(CAGs were) completely unfair in some ways, there should have carried on with exams, in a socially distanced manner or invigilated online - grades have to be earned not given’ (y13 Student 112).*

By March 2021, teachers and year 13 students were becoming aware of the corresponding arrangements for Summer 2021. ‘Teacher-assessed grades’ would be justified by performance in, usually, a variety of internally set and marked assessments taken in Spring/Summer 2021, potentially moderated by other centre-held data, and based only on the material actually covered. Overall, no year 13 students considered these worse than the centre-assessed grades used in 2020, typically expressing a high degree of confidence in teacher judgement, although about a third said they would have preferred the usual standard external papers. Teachers were less supportive, again evidencing a range of views, but with significant minorities expressing concerns about comparability across centres (34%) or pressure to inflate judgments made (23%).

Teachers and students typically talked about A Level grades in ‘exchange’ terms: what approaches would maintain ‘fairness’ of access to pathways with certain grades. Only 12% of teachers and 11% of students commented on the mathematical capacity reliably represented, and so their ‘use’ value: for example, on the impact of entering further work or study for which one was actually ill-prepared, despite the grades awarded.

## 3 Discussion and further research

This study contributes subject-specific knowledge of pre-university learning through the pandemic, including from students themselves. In a context where the new, aspirational, mathematics A Levels have not yet achieved equilibrium (Mason *et al.*, 2021) our data show that the pandemic has impeded progress towards that. Within distance learning as operationalized by teachers in this study, and the somewhat constrained in-centre practice adopted to accommodate pandemic-safer working, many newer and highly valued aspects of the specifications remained both marginalized and more difficult to teach and learn in meaning-making ways. In particular, the intended enhanced focus on problem solving, reasoning and proof, and on universal study of both mechanics and statistics, proved particularly problematic, and peer-collaborative approaches were found challenging to achieve in pandemic contexts.

These 16–18 year olds have usually struggled to learn mathematics effectively at a distance, even when teachers have significantly enhanced the ways in which they offer opportunities to learn. Further, a minority of students from a variety of backgrounds have continued to experience significant constraints on their productive working because of limited technological access. It is easy to imagine that these older, digital generation school and college students are likely to be minimally affected by such challenges, but this would appear not to be the case. Even where there are not technological or practical impediments, remote and isolated learning of mathematics makes very different cognitive, emotional and organizational demands from many of the digital activities with which these young people engage. Where there are financial or working space issues, families reluctant or unable to make additional or newer technical investment, or poor internet access, equitable solutions are not obvious. Limitations in access to work experience, to enrichment opportunities, to university visits and ‘widening participation’ opportunities in a pandemic, as well as ‘imposter syndrome’ consequences of cancelled examinations, are also likely to impact hardest on students from disadvantaged or under-represented backgrounds.

Our transition to university data contrast somewhat with e.g. Kinnear and Sangwin’s (2020), who showed little apparent impact on mathematical test functioning at entry in September 2020, although their data largely arise from Scottish students studying in a differently timed system. The contrasts between our teachers’ descriptions of provision for, and response from, year 12 students in March–July 2020 and those from students of their engagement with that provision, highlight how important it is, where possible, to research close to the target learner and learning, drawing on students’ lenses to complement those of teachers—and preferably, longitudinally. Such contrasts here might well arise from the challenges, also reported elsewhere (e.g. Royal Society and Joint Mathematical Council, 2021), of distinguishing remotely between attendance, engagement and learning.

However, the data also show a small minority of students valuing the less structured and more independent home learning experiences and many more appreciating the potential of such experiences to support later learning. Students and teachers also, over time, showed some development of their confidence to expand their technology-based functioning to support their learning, for example through peer ‘work groups’, using ‘whiteboards’ or ‘moving into breakout rooms’, but such developments took considerable time. Much of the work expected during periods of distance learning in this study, as in England more widely (Ofsted, 2021) was asynchronous, and a variety of structures within such provision was slower still to develop. Our study students recognized that asynchronous provision allowed them flexibility of timing (especially important if access to devices or internet speed are limited), as well as the opportunity to replay or pause the ‘lesson’—but they still very much valued synchronous opportunities to interact with teachers and peers.

Our initial analysis of the data around remote learning opportunity planned by teachers suggested that in the early months of the pandemic, teachers were frequently only beginning to learn how to structure for effective learning in the medium adopted. As a result, students’ in-depth engagement rapidly dwindled unless they were able to develop their own compensatory structures for learning. The exceptions were students in those three centres already making significant use of blended approaches to learning. However, teachers and students both reported progress with the range of such issues, via the expanded use of technological affordances, over time, and especially evident during the early 2021 home learning period. However, the constraints of technological access and domestic constraints during fulltime home learning continued to limit students’ access to enhanced remote provision. Throughout the pandemic to July 2021, it remained the case that the evidenced degree and detail of pandemic impact was highly variable by individual student. Economic or other disadvantage was often, but not straightforwardly, associated with aggravated impact.

The study has exposed important roles of high-stakes external examinations as motivator, catalyst for synthesizing learning, and perceived as ‘fair’ and ‘credible’. In the absence of such assessments, many students failed to fully capitalize on the learning they already had, and additionally, sometimes succumbed to ‘imposter syndrome’ because they felt the substitutes used lacked credibility. Indeed, the validity and reliability of teacher-sourced summative assessments remains an open question (Torrance, 2018). Going forward for the affected cohorts, it is important that learning opportunities in both pre-university and university settings offer alternative routes that support consolidation and synthesis of learning and credible affirmation of learning. This might also address a wider question, namely whether high-stakes examinations are themselves productive of an unhealthy dependence on them for catalysing productive learning. It is possible a continuous assessment or mixed mode of assessment might better support development of more sustainably constructive learning habits.

The persistent marginalization of new, widely valued, and possibly harder-to-teach aspects of the A Levels suggests that those values are not yet perceived to be fully reflected in influential drivers such as terminal assessments, so that it is also important to develop a range of ways to validate such learning. It is not obvious, for example, that the most effective way to assess what we value in problem solving, or statistical and data literacies, is through terminal written papers. Even for core mathematical functioning, moving to a wider assessment palette in these important pathways might have significant benefits.

The work reported here is in some depth but necessarily of a small sample, though as analysed above, there are grounds for confidence many of the findings are more widely applicable, at least to many A Level students elsewhere in the UK. It is clear that some of the evidenced impacts of the pandemic on learning, both negative and positive, are likely to have at least medium-term influence on student capacities. Going forward, it will be important to establish how the evidenced general and mathematics-specific affordances of technology to support learning can be harnessed and developed further over time, and some of the constraints addressed. It would also be helpful to learn from those contexts where more progress is being made. The subject-specific impacts on students’ learning, as well as on them more widely, including on their mental and emotional health, should be recognized and addressed: those are likely to affect progression for some time to come. The study has also focused attention on some constructive impacts of the examination system in catalysing synthesis of learning, and as trusted accreditation of that, while simultaneously bringing into focus some constraints of the current reliance in English mathematics assessment on terminal written papers. Findings suggest that a more mixed assessment economy might better support the development of highly valued aspects of mathematics functioning, as well as greater validity of some outcomes. Finally, our data point to a range of benefits of understanding pandemic impacts, positive as well as negative, at a subject-specific, as well as generic, level.

## Notes

**Jennie Golding** is an associate professor of mathematics education at UCL Institute of Education, London. Her background is in the teaching of mathematics from pre-school to university entrance level and in teacher initial and continuing education. Her research focuses on the interface between mathematics education policy and practice.
